# Risk of transmission of SARS-CoV-2 on international flights, a retrospective cohort study using national surveillance data in England

**DOI:** 10.1186/s12879-024-09052-2

**Published:** 2024-02-07

**Authors:** Joshua Howkins, Simon Packer, Eleanor Walsh, Deepti Kumar, Obaghe Edeghere, Matthew Hickman, Isabel Oliver

**Affiliations:** 1 UK Health Security Agency, 5th Floor, 10 South Colonnade, E14 4PU London, UK; 2 Health Protection Operations, UK Health Security Agency, 5th Floor, 10 South Colonnade, E14 4PU London, UK; 3 University of Bristol, Bristol, UK; 4 UK Health Security Agency, 61 Colindale Avenue, NW9 5EQ London, UK; 5 Population Health Sciences, Bristol Medical School, University of Bristol, Bristol, UK

**Keywords:** COVID-19, Transmission, Air travel, Contact tracing

## Abstract

**Background:**

It is not yet fully understood to what extent in-flight transmission contributed to the spread of Severe Acute Respiratory Syndrome Coronavirus 2 (SARS-CoV-2). This study aimed to determine the occurrence and extent of SARS-CoV-2 transmission in-flight and assess factors associated with transmission risk to inform future control strategies.

**Methods:**

Retrospective cohort study using data obtained from contact tracing of international flights arriving in England between 02/08/2021–15/10/2021. Transmission risk was estimated by calculating the secondary attack rate (SAR). Univariable and multivariable analyses of the SAR by specific risk factors was undertaken, including: number of in-flight index cases; number of symptomatic index cases; contact vaccination status; flight duration; proximity to the index case(s); contact age.

**Results:**

11,307 index cases linked to 667,849 contacts with 5,289 secondary cases reported. In-flight SAR was 0.79% (95% CI: 0.77–0.81). Increasing numbers of symptomatic cases (when > 4 index cases compared to one index case aOR 1.85; 95% CI: 1.40–2.44) and seating proximity to an index case (seated within compared to outside of two rows OR 1.82; 95% CI: 1.50–2.22) were associated with increased risk of secondary cases. Full vaccination history was protective (aOR 0.52; 95% CI: 0.47–0.57).

**Conclusions:**

This study confirms that in-flight transmission of SARS-CoV-2 occurred. There are factors associated with increased risk of infection. Contact tracing identified exposed persons who subsequently developed infection. A targeted approach to contact tracing passengers with the highest exposure risk could be an effective use of limited public health resources.

## Background

Following the emergence of Severe Acute Respiratory Syndrome Coronavirus 2 in December 2019 the virus rapidly spread globally, facilitated by international travel [1]. Travel-related interventions including contact tracing had previously been recognised as an important tool in limiting global transmission of other infectious diseases [2], leading to their implementation in many countries as a response to this new threat [1]. In the United Kingdom (UK), non-pharmaceutical interventions aiming to mitigate importation of cases through air travel included pre- and post-flight SARS-CoV-2 testing, contact tracing, and quarantine [3–5]. However, risk of transmission in-flight remained unclear [6], as did effective mitigation measures for airlines and other places of enhanced transmission risk [7], while the social and economic implications of some of the interventions on individuals and societies were highlighted [8].

Contact tracing was a key component of efforts to limit onward transmission of SARS-CoV-2. However, international strategies varied [1], drawing on the limited evidence from other infectious diseases [2, 9]. Contact tracing can be effective in reducing onward transmission of SARS-CoV-2 acquired in-flight [9], though timeliness and completeness are likely to be imperative to success [10–12]. To date, estimates of the risk of covid-19 transmission on international flights are based on small studies with findings that vary from no evidence of secondary transmission [6, 13], or low risk of transmission [3, 14–16], to high transmission risk [17]. Evidence from systematic reviews suggests closer seat proximity to index case, increasing number of index cases on flight, and non-complete vaccination status are factors positively associated with transmission risk, but also highlight the heterogeneity of findings [18–21] and over-reliance upon small samples which are more susceptible to bias and confounding.

Challenges to the public health response to the pandemic were encountered including in areas such as cross-border travel policy [5, 22]. Learning from the experience is required to inform the future response to new and emerging infectious diseases. In this retrospective study, we utilised a large national contact tracing dataset to determine the occurrence and extent of SARS-CoV-2 transmission on passenger aircraft and factors associated with transmission risk. We assess the utility of available genomic data to support inferences on transmission and describe contact tracing efforts to inform future public health control measures and policy.

## Methods

### Study population and data sources

This was a retrospective cohort study conducted using operational data collected in a standardised manner by Public Health England (PHE) and National Health Service (NHS) Test and Trace on COVID-19 cases who travelled on international flights during the study period and their contacts. The study considers a 74-day period of international flights arriving in England (02/08/2021 to 15/10/2021) with uniform data collection starting at the point when one data entry system was implemented, ending when passenger locator form data was no longer available to support contact tracing efforts. The study period also covers a time of recovering international travel [23], moderate background covid-19 incidence [24], and stable restrictions on passengers and airlines [25].

To travel to England in this period, passengers had to show a negative SARS-CoV-2 Polymerase Chain Reaction (PCR) test result (typically required within 72 h of their flight, though rules varied) prior to boarding and book PCR tests at days two and eight after landing. Positive tests were reported by laboratories to NHS Test and Trace. Completion rates for day two and eight tests were not available. Testing additional to those mandated at day two and eight also occurred, potentially in response to symptoms or for other individual reasons. Reports of positive test results prior to air travel are possible as passengers only had to show a negative test to board their plane. Index cases were defined as passengers with registered date of onset of symptoms or positive PCR test result between seven days before and two days after their international flight, implying these cases flew during their infectious period.

All included cases of COVID-19 had undergone contact tracing by NHS Test and Trace using a standardised questionnaire. Cases with a history of international flight travel were requested to provide flight details. If two or fewer positive cases in the same cabin (the section of an aircraft in which passengers travel) of a flight were identified in post-flight testing all passengers in that cabin were identified as contacts [26]. If more than two cases were on a flight, all passengers in the flight were defined as contacts. The study population includes all passengers defined as contacts on international flights arriving in England during the study period, and for whom contact tracing was undertaken. Contact tracing occurred in the same way as community contacts [27], though flight contacts were handled by a dedicated International Travel Contact Tracing (ITCT) team. Contact tracing would not have been undertaken if insufficient contact details were provided or their referral to the ITCT was not reached within the 24-hour cut off period for discarding referrals. Secondary cases were flight contacts with date of onset of symptoms or first positive SARS-CoV-2 PCR from three to 14 days after their flight exposure.

Datasets were an extract of international travel event data now held securely by the UK Health Security Agency (UKHSA). Genomic sequence data was linked where available from Covid-19 Genomic UK Consortium data also available from UKHSA using shared unique identifiers. Genomic sequencing from Pillar 2 (community) testing at the time followed near-random sampling from each region to the maximum coverage allowed by laboratory capacity [28]. During the study period roughly 15% of SARS-CoV-2 PCR positive samples were sequenced [28]. The dominant circulating variant of covid-19 during this time was Delta [28].

### Inclusion and exclusion criteria

#### Inclusion criteria

- all passengers and crew arriving by flight in England during the study period held on the international travel contact tracing datasets, having met case or flight contact definitions above.

Exclusion criteria:

- duplicate entry as defined by same flight number and same arrival date;
- sharing both Unique Property Reference Number (UPRN, a unique identifier for every addressable location in the UK) and genomic data between index and secondary case, as household transmission is more likely (if given UPRNs match, household sharing was assumed during infectious period);
- missing or void free-text data entry rendering data cleaning and linkage impossible.

### Data analysis

Transmission of covid-19 was quantified by calculating secondary attack rate (SAR) overall and for individual flights. The secondary attack rate is the probability of an infection occurring in a susceptible exposed group of people and can give an indication of how different risk factors or groupings relate to transmission risk. Genomic data was analysed for matches between flight-linked index and secondary case suggesting in-flight transmission. Only Single Nucleotide Polymorphism (0 SNP) matches were considered. Descriptive summary statistics were calculated, and we undertook univariable and multivariable logistic regression analyses using RStudio version 2202.02.0 to examine the relationship between transmission of SARS-CoV-2 (a binary yes/no outcome variable) risk and risk factors identified a priori. Exposure variables were categorised into clinically relevant groupings for number of index cases, (self-reported) vaccination status and age group, or as binary variables for seating proximity (within or outwith two seat rows of index case) and flight duration (less than or equal to two hours and greater than two hours). Symptomatic cases were defined by self-reported yes/no answer only.

Missing data [Table 1] was maintained, with the analysis using complete case analysis and no imputation. Exposure variables were sorted by percentage missing overall and stratified by key variables such as age, sex, and vaccination status. Upon visual inspection of the proportion missing by each characteristic, there was no obvious differential bias in missingness, and data were considered to be missing at random. Data on seat numbers was available for 2,670 secondary cases, most index cases, and no contacts. For this seating-known cohort, excluding instances of flights with more than one index case (leaving *n* = 1,553 secondary cases), we interrogated how many secondary cases were observed as sat within two rows of an index case. This was compared to how many might be expected to be sat within two rows if we assume random seating distribution on a plane of either the 3 × 3 or 3 × 4 × 3 seating configurations common to commercial flights. In the 3 × 3 configuration, an individual sat at random on a plane would be expected to be within two rows of a single index case 12.2% of the time. With the ‘3 × 4 × 3’ configuration common to longer haul flights, 8.1% of randomly sat individuals would expect to be sat within two rows of a single index case. These estimated figures of seating proximity were compared to the observed data for how often secondary cases were sat next to index cases to calculate odds ratios.

**Table 1:** missing data summary for key characteristics in datasets. Contacts only column does not contain secondary cases for ease of comparison. UPRN = Unique Property Reference Number

Exposure variable	Index cases *n* = 11,307	Contacts only *n* = 662,560	Secondary cases *n* = 5,289
	Missing data, n = (%)		
Whether symptomatic or not	0 (0)	Not applicable	Not required
Age	0 (0)	0 (0)	0 (0)
Vaccination status	1,316 (12)	262,354 (40)	2,145 (41)
Sex	6,849 (61)	575,179 (87)	4,424 (84)
Seat number	1,757 (16)	662,560 (100)	2,619 (50)
Flight duration	Not required	599,275 (90)	4,897 (93)
Genomic data	8,379 (74)	Not applicable	4,526 (86)
UPRN	2,559 (23)	114,341 (22)	1,003 (19)

Complex survey design using the survey package (version 4.2-1) in RStudio accounted for clustering of data by flight instance using flight number and date of travel to generate date-specific flight numbers. The impact of clustering of data was assessed for both univariable and multivariable logistic regressions and is presented in the results with robust standard errors. Variables’ contributions to the regression models were assessed using chi-squared test, improvement in Akaike Information Criterion, and likelihood ratio tests. The inclusion of both the number of index cases and the number of symptomatic cases on flights improved the fit of the model (Chi-squared 17.8, *p* < 0.001) with only moderate co-linearity, as measured by variance inflation factor, for number of index cases and number of symptomatic cases [29]. Interactions between variables were considered by sequential addition to the model; only age of contacts was considered a confounding variable.

## Results

### Descriptive epidemiology

**Fig. 1:**
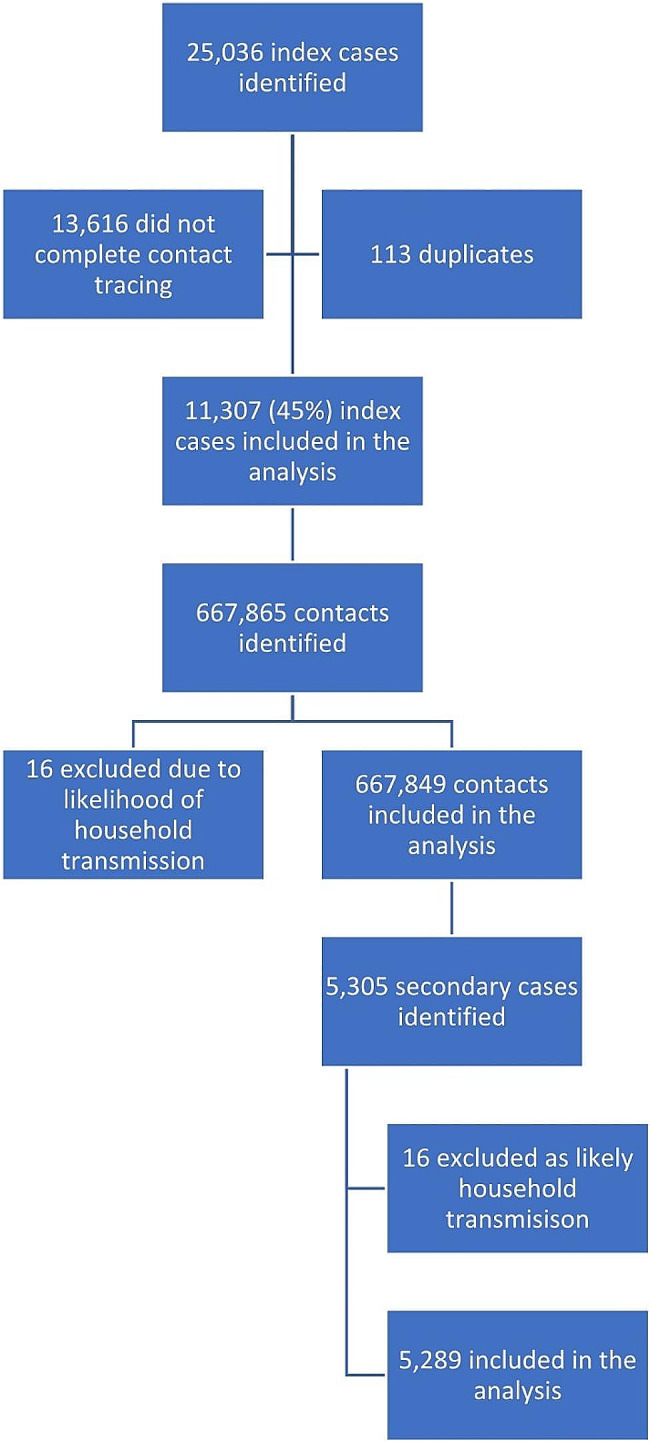
Flow diagram showing population of cases, their contacts, and secondary cases used in the study

**Fig. 2:**
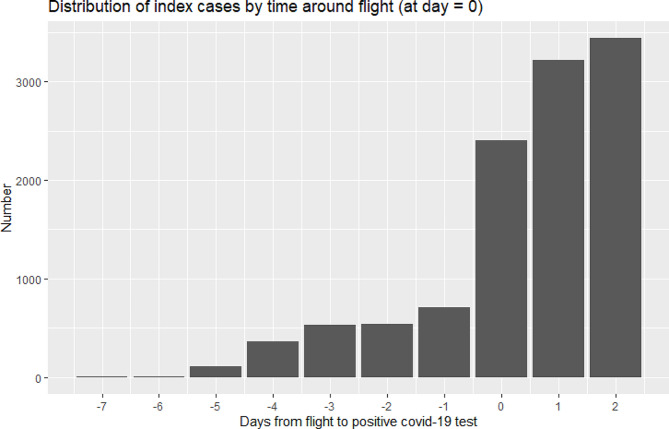
Distribution of SARS-C0V-2 detection time (first positive PCR test of contact traced index cases) around flight (day zero)

Index case dataset initially included 25,036 individuals flying during study period [flow diagram Fig. 1]. Screening removed 13,616 (54%) who were not contact traced and 113 duplicates, leaving *n* = 11,307. These index cases arose from 7,831 different flights. The total number of flights arriving in England in that period is not publicly available. Index case distribution on individual flights was positively skewed: median number of index cases on a flight was *n* = 1 (mean = 1.3, range = 1–18). Most index cases tested positive on the day of flight or within two days after [Fig. 2].

There were 667,865 flight contacts of these cases identified. 16 were removed per exclusion criteria for sharing both UPRN and genomic data with an index case, leaving *n* = 667,849 [Fig. 1]. Flights held a median of 83 contacts (mean 85, range 1-343).

**Fig. 3:**
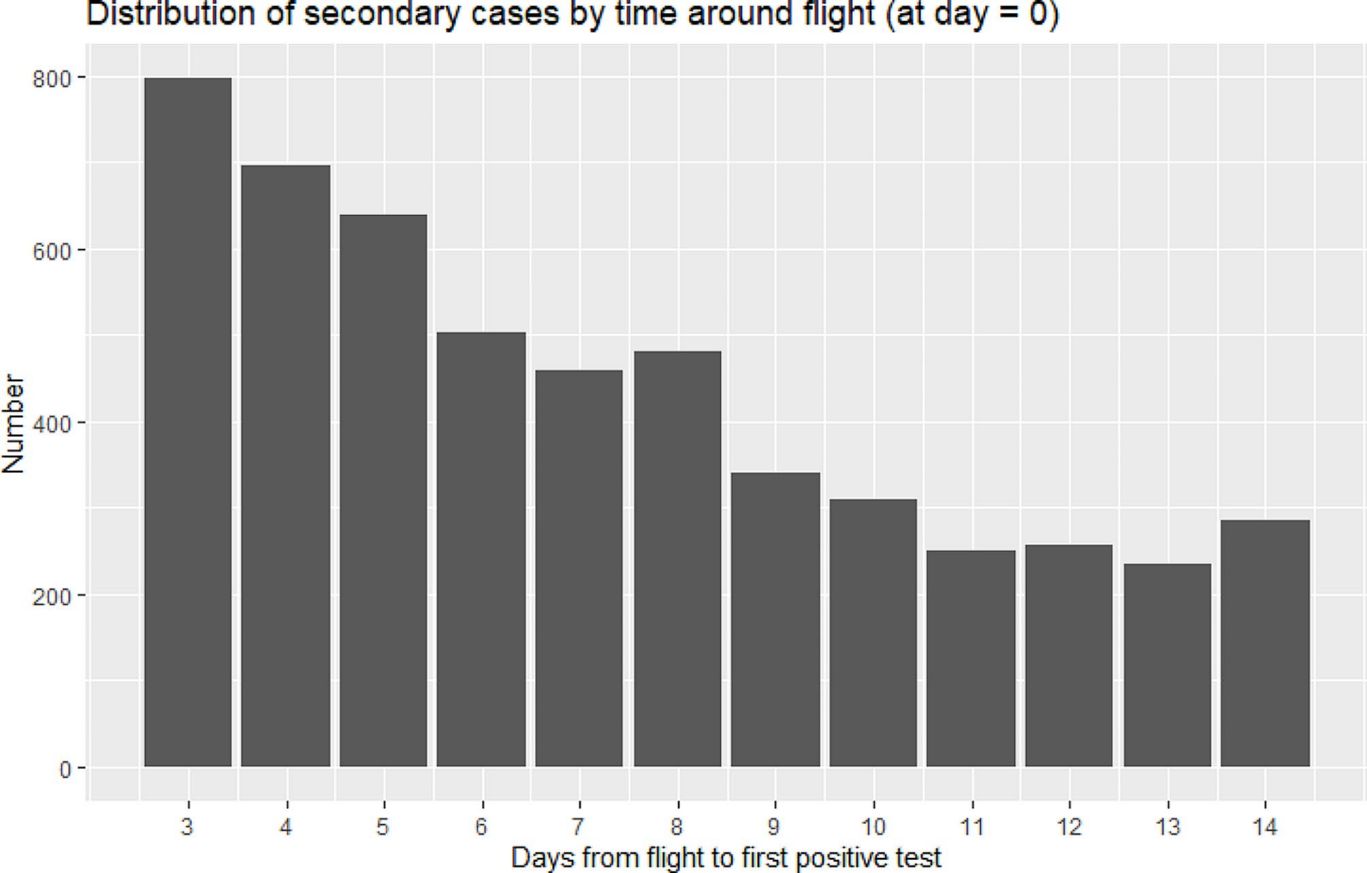
Distribution SARS-C0V-2 detection time (first positive PCR test of secondary cases) around flight (at day zero, not seen)

The secondary case dataset included 5,305 individuals from the 667,849 contacts; 16 were removed after applying exclusion criteria, leaving *n* = 5,289 [Fig. 1]. Distribution of secondary cases per flight was positively skewed, median = 0 (mean = 0.7, range = 0–13). The number of days from when secondary cases testing positive since flight followed a Poisson distribution, decreasing in rate after the first few days [Fig. 3]. An increase is noted at day eight, likely due to post-flight-testing rules.

### Demographics

Contact traced and non-contact traced individuals (not included in the analysis) were compared for difference in characteristics to consider selection bias [Table 2]. Traced individuals were older than non-traced (mean age 41 vs. 38 years, t-test *p* < 0.001) with a slightly higher ratio of males to females in the traced group. Traced individuals were more likely to be fully vaccinated [Table 2].

**Table 2:** Differences between traced and untraced index case populations and contact and secondary case populations for key characteristics. Key: ^1^ mean, median (IQR); ^2^ Welch two sample t-test, Pearson’s Chi-squared test

Characteristic		Traced *n* = 13,616 (%)	Not traced *n* = 11,307 (%)	p value ^2^	Contacts only *n* = 662,560 (%)	Secondary cases *n* = 5,289 (%)	p value ^2^
Age		38, 37 (22, 52)^1^	41, 43 (27, 56) ^1^	< 0.001	44, 43 (30, 56)^1^	46, 46 (33, 57) ^1^	< 0.001
Sex				< 0.001			< 0.001
	Female	2,256 (17)	2,130 (19)		42,052 (6)	389 (7)	
	Male	2,408 (18)	2,328 (21)		45,329 (7)	476 (9)	
	Unknown	8,952 (66)	6,849 (61)		575,179 (87)	4,424 (84)	
Number of vaccinations				< 0.001			< 0.001
	None	2,414 (18)	1,292 (11)		50,034 (7.6)	604 (11)	
	Partially (one)	850 (6.2)	398 (3.5)		18,725 (2.8)	166 (3.1)	
	Fully (two)	8,133 (60)	8,301 (73)		331,447 (50)	2,374 (45)	
	Unknown	2,219 (16)	1,316 (12)		262,354 (40)	2,145 (41)	

Secondary cases differed from the contacts only group [Table 2]. Secondary cases were older (mean age 46 vs. 44 years, t-test *p* < 0.001), more likely to be unvaccinated (11% vs. 7.6%), and less likely to have received two vaccinations against COVID-19, termed as ‘fully vaccinated’ for the time (45% vs. 50%, Chi-squared test for difference between groups *p* < 0.001).

### In-flight transmission risk

The overall secondary attack rate (SAR) in our study population was 0.79% (95% CI: 0.77 to 0.81; 5,289 cases from 667,849 contacts). Stratified by individual flight, the mean SAR = 0.80% (minimum 0%, median 0%, maximum 25%).

Genomic data were available for 2,298 (26%) index cases and 763 (14%) secondary cases. However, information was available for cases linked by flight exposure with available UPRN data in only 173 instances. Of these, 26 (15%) shared genomic data without a matching UPRN number (indicating no household sharing but linked transmission). There were four instances (2.3%) of shared UPRN without genomic match and 143 (83%) with neither UPRN nor genomic match. The extent of this missing data meant that SAR was not calculated in this subset.

### Exposure variable analysis

Flight duration information was present for 63,677 (9.6%) contacts and 392 (7.4%) secondary cases. There was weak evidence that the number of secondary cases was slightly higher on longer flights of durations longer than two hours against those less than or equal to two hours, odds ratio (OR 1.19, 95% CI: 0.97 to 1.46; *p* = 0.10) [Table 3]. More secondary cases were observed to be sat within two rows of an index case than expected in a randomly distributed sample in both seating scenarios: in ‘3 × 4 × 3’ seating configuration (OR 2.86, 95% CI: 2.29 to 3.56; *p* < 0.001) and in the ‘3 × 3’ seating configuration (OR 1.82, 95% CI: 1.50 to 2.22; *p* < 0.001) [Table 3]. There was strong evidence that the secondary cases were lower among vaccinated individuals (OR 0.59, 95% CI: 0.54 to 0.65; *p* < 0.001) [Table 3]. The odds of finding secondary cases were higher in older contact age groups: 40–64 years (OR 1.39, 95% CI: 1.27 to 1.53; *p* < 0.001) and greater than 65 years (OR 1.32, 95% CI: 1.18 to 1.48; *p* < 0.001), compared to the youngest age group (0–24 years) [Table 3].

**Table 3:** Above: Results, secondary attack rates and odds ratios for studied exposure variables (aOR given where applicable). Table key: UVA = univariable analysis; MVA = multivariable analysis; SAR = secondary attack rate; OR = odds ratio; aOR = adjusted odds ratio; CI = Confidence Interval; Ref. = reference row for that section. Blank results space implies data not applicable; flight duration and proximity were not included in the multivariable regression model. Explanation of seating configuration assumptions in methods

					UVA without clustering			UVA clustered by flight			MVA without clustering			MVA clustered by flight		
Risk factor		**Contacts only (n)**	**Secondary cases (n)**	**SAR (%)**	**OR**	**95% CI**	**p** **value**	**OR**	**95% CI**	**p** **value**	**aOR**	**95% CI**	**p value**	**aOR**	**95% CI**	**p value**
Number of index cases on plane	**1**	460,837	3,206	0.69	Ref.	Ref.	Ref.	Ref.	Ref.	Ref.	Ref.	Ref.	Ref.	Ref.	Ref.	Ref.
	**2–3**	170,528	1,534	0.89	1.29	1.22, 1.37	< 0.001	1.29	1.20, 1.40	< 0.001	1.17	1.05, 1.29	0.003	1.17	1.04, 1.31	0.01
	**> 4**	31,195	549	1.73	2.53	2.31, 2.77	< 0.001	2.53	2.23, 2.87	< 0.001	1.59	1.27, 1.99	< 0.001	1.59	1.24, 2.06	< 0.001
Number of symptomatic index cases on plane	**0**	245,151	1,578	0.64	Ref.	Ref.	Ref.	Ref.	Ref.	Ref.	Ref.	Ref.	Ref.	Ref.	Ref.	Ref.
	**1**	304,176	2,375	0.77	1.21	1.14, 1.29	< 0.001	1.21	1.12, 1.31	< 0.001	1.16	1.07, 1.26	< 0.001	1.16	1.06, 1.27	0.001
	**2–3**	95,590	987	1.02	1.60	1.48, 1.74	< 0.001	1.60	1.45, 1.78	< 0.001	1.22	1.06, 1.40	0.004	1.22	1.04, 1.43	0.01
	**> 4**	17,643	349	1.94	3.07	2.73, 3.45	< 0.001	3.07	2.61, 3.62	< 0.001	1.85	1.40, 2.44	< 0.001	1.85	1.32, 2.58	< 0.001
Vaccination status of contacts	**Not vaccinated**	50,034	604	1.19	Ref.	Ref.	Ref.	Ref.	Ref.	Ref.	Ref.	Ref.	Ref.	Ref.	Ref.	Ref.
	**Partially vaccinated**	18,725	166	0.88	0.73	0.62, 0.87	< 0.001	0.73	0.62, 0.87	< 0.001	0.75	0.63, 0.89	< 0.001	0.75	0.63, 0.89	< 0.001
	**Fully vaccinated**	331,447	2,374	0.71	0.59	0.54, 0.65	< 0.001	0.59	0.53, 0.66	< 0.001	0.52	0.47, 0.57	< 0.001	0.52	0.46, 0.57	< 0.001
	**Unknown**	262,354	2,145	0.81	0.68	0.62, 0.74	< 0.001	0.68	0.60, 0.76	< 0.001						
Age group of contact (years)	**0–24**	82,262	543	0.66	Ref.	Ref.	Ref.	Ref.	Ref.	Ref.	Ref.	Ref.	Ref.	Ref.	Ref.	Ref.
	**25–39**	212,538	1,406	0.66	1.00	0.91, 1.11	> 0 0.9	1.00	0.90, 1.12	> 0.9	1.05	0.93, 1.20	0.4	1.05	0.93, 1.19	0.4
	**40–65**	295,008	2,707	0.91	1.39	1.27, 1.53	< 0.001	1.39	1.24, 1.55	< 0.001	1.49	1.32, 1.68	< 0.001	1.49	1.32, 1.68	< 0.001
	**> 65**	72,752	633	0.87	1.32	1.18, 1.48	< 0.001	1.32	1.14, 1.53	< 0.001	1.49	1.27, 1.74	< 0.001	1.49	1.27, 1.74	< 0.001
Flight duration	**<= 2 h**	29,168	164	0.56	Ref.	Ref.	Ref.									
	**> 2 h**	34,117	228	0.66	1.19	0.97, 1.46	0.1									
Proximity (seated within two rows)	**Estimated 3 × 3 configuration**	1,364	189		Ref.	Ref.	Ref.									
	**Observed**	1,240	313		1.82	1.50, 2.22	< 0.001									
	**Estimated 3 × 4 × 3 configuration**	1,427	126		Ref.	Ref.	Ref.									
	**Observed**	1,240	313		2.86	2.29, 3.56	< 0.001									

A higher number of index cases on a flight was associated with increased risk of secondary cases. Taking one index case as the reference, instances where two to three index cases were reported had an OR of finding secondary cases post flight of 1.29 (95% CI: 1.22 to 1.37; *p* < 0.001), and those with greater than four index cases an OR 2.53 (95% CI: 2.31 to 2.77; *p* < 0.001) [Table 3]. The number of symptomatic index cases was also associated with higher risk of secondary cases, and highest when four or more cases had been reported (taking zero cases as reference, OR 3.07, 95% CI: 2.73 to 3.45; *p* < 0.001) [Table 3].

Adjusted analysis [Table 3] showed strong evidence that the number of symptomatic cases on the flight was associated with increased risk of secondary cases after adjusting for other independent predictors: with one reported symptomatic case aOR 1.16 (95% CI: 1.07 to 1.26; *p* < 0.001); with two to three symptomatic cases aOR 1.22 (95% CI: 1.06 to 1.40; *p* = 0.004); and when more than four symptomatic cases were reported aOR 1.85 (95% CI: 1.40 to 2.44; *p* < 0.001).

Increasing number of index cases remained associated with increasing secondary cases within the multivariable model, with instances of greater than four index cases having an aOR 1.59 (95% CI: 1.27 to 1.99; *p* < 0.001) compared to reference of one index case. Vaccination status of contacts remained strongly associated with risk of secondary cases, with partial vaccination conferring an aOR of 0.75 (95% CI: 0.63 to 0.89; *p* < 0.001) and full vaccination aOR 0.52 (95% CI: 0.47 to 0.57; *p* < 0.001). Flight duration and proximity reduced power of the model and were removed. Calculation of robust standard errors to account for the clustered nature of data by flight did not change significance levels met for any variable.

## Discussion

The overall in-flight secondary attack rate was 0.79%. This attack rate is substantially lower than that reported in the UK amongst household contacts of 10.8%, and non-household contacts of 3.7% [30]. This may be explained by differences in contact definition as well as other factors such as duration of contact and air quality measures in flights. This finding supports available literature regarding in-flight secondary attack rates [14, 18–20] whilst strengthening previous results that may have been underpowered due to small sample size [17].

Secondary cases were more likely to be identified with increased number of index cases on a plane, particularly if these are symptomatic. Vaccination was also a strong protective factor of in-flight transmission. These associations remain true after adjustment within the multivariable model for number of index cases, number of symptomatic cases, vaccination status, and age, and when accounting for clustering at flight level. Secondary cases were more likely to be found within two rows of an index case. The findings are consistent with those reported in other studies [14, 18–20], and highlight the importance of number of index cases, proximity, and vaccination status. Flight duration is shown in our data to potentially be less significant a risk factor than in earlier studies [14, 19], though a paucity of longer flights meant that duration was not able to be measured in more granular detail. These studies consider earlier periods in the pandemic, and the difference observed may be due to risk mitigation strategies required to be brought in by airlines by the time of our study including those aiming to reduce passenger movement in-flight, changes in dominant variant, changes in use of personal protective measures, and increased prevalence of vaccination [26].

Genomic sequencing data strongly confirmed the evidence of SARS-CoV-2 transmission onboard flights, by showing unrelated cases with genetically identical sequences, and is a potentially powerful tool available to public health policy makers. However, unfortunately, despite the relatively high sequencing rates in the UK [28], coverage, together with poor quality data on place of residence, were not sufficient to assess transmission.

The strengths of this study lie in the unique national dataset affording high power and multivariable analysis of risk factors. However, contact tracing was completed for only 45% (11,307/25,193) of the eligible index cases in this cohort due to inadequate information provided by travellers and the very high caseload of the team undertaking contact tracing, which likely also induced the high proportion of missingness for some variables. Although public health advice was given to 679,172 identified cases and contacts (11,307 index cases and 667,865 contacts), a large proportion of the population at risk will have been missed and not received additional advice or intervention related to their flight exposure. The proportion of booked PCR tests that were taken ineffectively or not completed at all is unknown and the traced individuals in our study tended to be older and more likely to be vaccinated (known protective factors), potentially underestimating the true SAR. Measurement bias in defining the in-flight aspect of transmission was attempted to be investigated using genomic data; however, data were not available to achieve this. Therefore, we assumed transmission within this defined cohort occurred on flights, though, in some cases, it could have occurred at another time during the infectious period, from alternative index cases. Although our study population is large, it occurred over a short period, and behaviour and other changes at different times during the pandemic would likely alter some findings. Our study was not able to determine the effect of flight duration, likely due to substantial missing or free-text field input. The assessment of seating proximity risk had limitations including assuming that in the estimated comparator samples, individuals would be randomly seated not clustered in groups, and in comparing only to the observed cohort where a single index case was observed on a flight. Missing data impacts the power of our study but the effects of this are offset by the large dataset. No systematic bias was observed in the distribution of missing data when stratified by key variables.

This study adds to the body of evidence of the value of contact tracing of in-flight contacts and identifies factors that may be used to develop a targeted approach to contact tracing of flights to secure maximum public health value particularly when resources are constrained. This is likely to improve the effectiveness of contact tracing while reducing any adverse impact of quarantining passengers with minimal or no risk of infection following exposure. However, the analysis is context-specific as factors including covid-19 variant, background prevalence, and enforced enhanced hygiene measures changed with time and limit generalisation. Further work is required to understand how transmission changed in association with variation of these factors to provide further evidence to inform future policy.

## Conclusions

In conclusion, this study provides evidence of transmission of SARS-CoV-2 on international passenger flights and identifies factors associated with increased risk of secondary transmission. The large absolute number of secondary cases observed during the study period indicate this may be an important route of transmission and public health interventions should be considered to help control this in future epidemics. The scale of contact tracing of flight contacts was unprecedented but the effectiveness of contact tracing in the study period was potentially limited by poor quality information and lack of completeness in contacting potentially at-risk individuals. Despite this, large numbers of contacts and secondary cases were identified and isolated, therefore potentially limiting some onward spread. Therefore, efforts should be made to improve the information available to support contact tracing, such as through improved data sharing with public health authorities. The risk factors identified suggest that a targeted approach to contact tracing may be effective, particularly where public health capacity is constrained.

## Data Availability

The datasets generated and/or analysed during the current study are not publicly available as they were collected as part of a public health response and are considered sensitive. Access to anonymised data will be considered by the corresponding author on reasonable request.

## Abbreviations

ITCT: International Travel Contact Tracing
NHS: National Health Service
OR: odds ratio
PCR: Polymerase Chain Reaction
PHE: Public Health England
SAR: Secondary attack rate
SARS-CoV-2: Severe Acute Respiratory Syndrome Coronavirus 2
0 SNP: Single Nucleotide Polymorphism
UPRN: Unique Property Reference Number
UKHSA: United Kingdom Health Security Agency
