# Correction: Risk of transmission of SARS-CoV-2 on international flights, a retrospective cohort study using national surveillance data in England

**DOI:** 10.1186/s12879-024-09182-7

**Published:** 2024-04-04

**Authors:** Joshua Howkins, Simon Packer, Eleanor Walsh, Deepti Kumar, Obaghe Edeghere, Matthew Hickman, Isabel Oliver

**Affiliations:** 1 UK Health Security Agency, 5th Floor, 10 South Colonnade, E14 4PU London, UK; 2 Health Protection Operations, UK Health Security Agency, 5th Floor, 10 South Colonnade, E14 4PU London, UK; 3 NIHR Health Protection Research Unit in Behavioural Science and Evaluation, University of Bristol, Bristol, UK; 4 UK Health Security Agency, 61 Colindale Avenue, NW9 5EQ London, UK; 5 Population Health Sciences, Bristol Medical School, University of Bristol, Bristol, UK

Following publication of the original article [1], we have been notified of the below corrections:

## Acknowledgements

Isabel Oliver, Matt Hickman and Eleanor Walsh are members of the NIHR Health Protection Research Unit in Behavioural Science and Evaluation at University of Bristol.

## Funding

Joshua Howkins holds an NIHR Academic Clinical Fellowship (ACF-2022-13-013).

## Affiliations

The affiliations for Eleanor Walsh and Mattew Hickman are: Population Health Sciences - Bristol Medical School & NIHR Health Protection Research Unit in Behavioural Science and Evaluation at University of Bristol. Isabel Oliver is affiliated with NIHR Health Protection Research Unit in Behavioural Science and Evaluation at University of Bristol, along with her UKHSA affiliation.
